# Exploring positive and negative body image and health-related quality of life in women with endometriosis: a latent profile analysis

**DOI:** 10.1093/humrep/deaf127

**Published:** 2025-07-01

**Authors:** Sara Iannattone, Martina Rapisarda, Gioia Bottesi, Silvia Cerea

**Affiliations:** Department of General Psychology, University of Padova, Padova, Italy; Department of General Psychology, University of Padova, Padova, Italy; Department of General Psychology, University of Padova, Padova, Italy; Department of General Psychology, University of Padova, Padova, Italy

**Keywords:** endometriosis, chronic illness, body image, health-related quality of life, latent profile analysis

## Abstract

**STUDY QUESTION:**

What are the profiles of body image (both negative and positive) and their associations with health-related quality of life (HRQoL) and endometriosis-related symptoms in women with endometriosis?

**SUMMARY ANSWER:**

Three distinct body image profiles were identified, which significantly differed in HRQoL dimensions and both number and types of endometriosis-related symptoms.

**WHAT IS KNOWN ALREADY:**

Endometriosis is a chronic health condition characterized by multiple symptoms, which lead to a diminished HRQoL. Body image is a critical concern for women with endometriosis due to the impact of the illness and its treatments on their bodies.

**STUDY DESIGN, SIZE, DURATION:**

This cross-sectional study involved 270 Italian women who self-reported a diagnosis of endometriosis. They were recruited through the social media pages of Italian endometriosis organizations between March and September 2023.

**PARTICIPANTS/MATERIALS, SETTING, METHODS:**

The mean age of the participants was 36.4 years (SD = 7.46, range = 18–56), while the mean time since diagnosis was 347 months (SD = 80). The main endometriosis diagnosis was deep endometriosis (58.1%), and the main method of diagnosis was a clinical method (70.7%). The participants completed a socio-demographic and medical history schedule, as well as the following self-report questionnaires: Functionality Appreciation Scale, Body Appreciation Scale-2, Endometriosis Health Profile-30, and Body Image Scale. Latent Profile Analysis (LPA), ANOVA, and chi-square tests were employed to analyze the data. In particular, given the data-driven nature of LPA, no *a priori* hypotheses were formulated regarding the number or pattern of the profiles.

**MAIN RESULTS AND THE ROLE OF CHANCE:**

The LPA revealed three profiles: ‘Low body appreciation and strong body dissatisfaction’ (47.8%), ‘Strong body appreciation and low body dissatisfaction’ (17%), and ‘Moderate body appreciation and body dissatisfaction’ (35.2%). ANOVA showed differences in all HRQoL dimensions and number of endometriosis-related symptoms among profiles, with women in the ‘Strong body appreciation and low body dissatisfaction’ profile exhibiting better HRQoL and fewer endometriosis-related symptoms compared to the other profiles (*P*<0.001). Finally, chi-square tests revealed that participants in the ‘Low body appreciation and strong body dissatisfaction’ profile were significantly more likely to report painful and a-specific symptoms compared to participants in the other profiles.

**LIMITATIONS, REASONS FOR CAUTION:**

The study’s cross-sectional design precludes any conclusions about causality. Furthermore, the absence of a control group of women without endometriosis makes it unclear whether the identified body image profiles are specific to endometriosis or represent broader patterns in the general population. Also, since LPA is inherently exploratory, these results offer only preliminary insights into how negative and positive body image may interact and relate to HRQoL and endometriosis-related symptoms in women with endometriosis. Additionally, the use of self-referral through endometriosis organizations, combined with the absence of sexuality-based demographics and other potentially relevant psychological and biological variables, may limit both the generalizability and the comprehensiveness of our results. Finally, the exclusive use of self-report questionnaires, which are subject to biases, and the inclusion of a small proportion of participants who reported seeking psychological consultation for a possible eating disorder may have influenced the results.

**WIDER IMPLICATIONS OF THE FINDINGS:**

Positive body image may act as a protective buffer against negative HRQoL outcomes and these results may be useful for developing psychological interventions aimed at promoting psychological and physical well-being in women with endometriosis.

**STUDY FUNDING/COMPETING INTEREST(S):**

This research received no specific funding. The authors report no conflicts of interest.

**TRIAL REGISTRATION NUMBER:**

N/A.

## Introduction

Endometriosis is a chronic health condition characterized by the presence of endometrium-like tissue outside its typical region, the uterine cavity, affecting ∼10% of women of reproductive age (Zondervan *et al.*, 2018, 2020). Women with endometriosis suffer from multiple and heterogeneous symptoms, including chronic pelvic pain, dysmenorrhea (menstrual pain), dyspareunia (painful sexual contact), and dyschezia (defecation pain). The diagnosis of endometriosis is often overlooked (Harzif *et al.*, 2024; Lampl *et al.*, 2025), resulting in a diagnostic delay that varies widely across studies and population samples (ranging from 1.5 to 11.4 years; for a review, see Fryer *et al.*, 2025).

Endometriosis negatively affects several aspects of women’s lives (Young *et al*., 2015), leading to a diminished health-related quality of life (HRQoL). This includes physical symptoms that interfere with functioning and daily activities (Hunsche *et al.*, 2023), social impairments (Culley *et al.*, 2013; Rush and Misajon, 2018), and psychological symptoms, with anxiety, depressive symptoms, and poor body image being among the most prevalent (Chen *et al.*, 2016; Sullivan-Myers *et al.*, 2021; van Barneveld *et al.*, 2022).

### Body image in the context of endometriosis

Body image has emerged as a critical concern for women with endometriosis given the impact of the illness and its treatment on their bodies (e.g. abdominal swelling, surgical scarring; Sayer-Jones and Sherman, 2021; Pehlivan *et al.*, 2024; Mills *et al.*, 2025). Although there is currently no definitive cure for endometriosis, treatment commonly involves repeated surgical procedures and hormone therapy aimed at slowing disease progression and alleviating pain (Becker *et al.*, 2017; Casper, 2017). These treatments, however, often cause unwanted side effects, including visible changes in physical appearance such as scarring and weight gain (Mills *et al.*, 2025). Beyond these visible changes, endometriosis and its treatment also result in less observable functional impairments, such as subfertility (La Rosa *et al.*, 2020) and diminished bodily control, which can reduce women’s sense of agency (Mills *et al.*, 2025).

Taken together, these bodily changes deeply impact body image in women with endometriosis (Sayer-Jones and Sherman, 2023; Pehlivan *et al.*, 2024). Consequently, many individuals with this condition report a negative relationship with their bodies, often describing them as defective, broken, damaged, or unfamiliar (Van Niekerk *et al.*, 2022b). This negative relation with the body has been identified as a significant correlate of lower physical and emotional HRQoL and has also been linked to dysmenorrhea and higher levels of endometriosis-related pain (Melis *et al.*, 2015; Van Niekerk *et al.*, 2022b). On the other hand, a positive relation with the body (i.e. positive body image) has been associated with reduced endometriosis-related pain (Volker and Mills, 2022).

Positive body image is a multidimensional construct that encompasses an overarching respect and appreciation for the body and its functionality (Tylka and Wood-Barcalow, 2015a; Wood-Barcalow *et al.*, 2010). Among its components, positive body image includes body and functionality appreciation. Body appreciation refers to accepting and respecting one’s body (Tylka and Wood-Barcalow, 2015a) and represents a protective factor for both physical and mental health (Linardon *et al.*, 2022, 2023; Cerea *et al.*, 2024, 2025; Mancin *et al.*, 2024). Functionality appreciation, on the other hand, refers to appreciating, respecting, and honoring the body for what it is capable of doing (Alleva and Tylka, 2021).

Both body and functionality appreciation are likely to be important aspects of body image in the context of endometriosis, given the need of these women to live with extensive physical changes and bodily impacts (Mills *et al.*, 2025). However, few studies have investigated positive body image in the context of endometriosis (e.g. Sayer-Jones and Sherman, 2021; Volker and Mills, 2022; Sullivan-Myers *et al.*, 2023; Grano *et al.*, 2025; Mills *et al.*, 2025), as most research conducted so far has focused on negative body image. However, the qualitatively different constructs of positive and negative body image (Tylka and Wood-Barcalow, 2015a) suggest that measuring only a single aspect or dimension (e.g. positive or negative) of body image may provide an incomplete picture of body image in the context of endometriosis.

Studies on positive body image in women with endometriosis have shown that accepting one’s body and its functionality is associated with reduced pain (Volker and Mills, 2022) and enhanced coping (Sayer-Jones and Sherman, 2021, 2023), providing evidence for a potential protective role of positive body image in improving HRQoL. Despite these promising findings, as previously highlighted, most research in this area rarely includes measures of positive body image or examines its relationship with HRQoL.

Existing studies have also often failed to assess HRQoL in relation to endometriosis-specific symptoms. General HRQoL instruments, such as the Short Form Health Survey-36 (SF-36), are commonly used in this context, but do not address key issues relevant to endometriosis, such as dyspareunia and dysmenorrhea (Shum *et al.*, 2018; Flaxman *et al.*, 2020; Schneider *et al.*, 2020). In contrast, disease-specific tools are more sensitive to the lived experience of patients, as they include items developed from direct input of patients (Bourdel *et al.*, 2019).

### The current study

To fill the gaps of previous research, the current study aimed to identify relevant profiles of body image (both positive and negative) in women with endometriosis using an exploratory, person-centered approach (i.e. Latent Profile Analysis, LPA). In contrast to variable-centered approaches (e.g. regression), which focus on associations between variables across the full sample, person-centered statistical techniques identify relatively homogeneous subgroups of individuals based on combinations of variables (Howard and Hoffman, 2018).

In particular, LPA is considered effective in preserving the complexity and heterogeneity of individual profiles, proving useful for addressing clinically relevant topics (Ribeiro da Silva *et al.*, 2019). Thus, this approach is particularly well-suited for exploring complex, multidimensional constructs like body image, which may manifest differently across individuals. However, given the hypothesis-generating nature of LPA, no *a priori* assumptions were made regarding the number or pattern of latent profiles. Rather, this preliminary analysis aimed to uncover naturally occurring profiles in the data, which could inform future theory-driven investigations.

Finally, we also sought to compare the identified profiles in terms of HRQoL dimensions specific to endometriosis, using a disease-specific instrument (i.e. Endometriosis Health Profile-30; Jones *et al.*, 2001), as well as the total number and types of endometriosis-related symptoms. Specifically, endometriosis symptoms were grouped into three categories: non-painful symptoms due to menstrual cycle alterations (i.e. menorrhagia, metrorrhagia, menstrual spotting, and irregular periods), painful symptoms (i.e. pelvic pain, dyspareunia, dyschezia, dysmenorrhea, and vulvodynia), and a-specific symptoms (i.e. weight gain, reduced sexual desire, abdominal bloating, and headache). Based on previous variable-centered research (e.g. Melis *et al.*, 2015; Van Niekerk *et al.*, 2022a,b; Volker and Mills, 2022), we anticipate that profiles characterized by high levels of negative body image will be associated with poor HRQoL and elevated endometriosis symptoms, and profiles characterized by high levels of positive body image will be associated with better HRQoL and fewer endometriosis symptoms.

## Materials and methods

### Participants and procedure

A total of 270 Italian participants who were assigned female at birth, self-identified as women, and self-reported having received a surgical or clinical diagnosis of endometriosis took part in the study. The participants reported a mean age of 36.4 years (SD = 7.46, range = 18–56), a mean education of 14.8 years (SD = 3.41, range = 5–21), and a mean Body Mass Index (BMI) of 23.4 (SD = 4.58, range = 16.38–44.06). Concerning the time since endometriosis diagnosis, the mean time was 347 months (SD = 80).

Participants were recruited through the social media pages (Facebook and Instagram) of Italian endometriosis organizations between March and September 2023. They were invited to take part in a study investigating HRQoL in women with endometriosis. The inclusion criteria for the study were: (i) a clinical diagnosis of endometriosis, established through a comprehensive clinical evaluation conducted by a physician, supported by instrumental examinations (e.g. transvaginal ultrasound or other diagnostic imaging techniques) or a surgical diagnosis of endometriosis; (ii) assigned female at birth; and (iii) age 18 years or older. The exclusion criterion was a current diagnosis of another persistent health condition not related to endometriosis. Specifically, participants were asked to report any current medical conditions via a screening question included at the beginning of the survey. Those who indicated the presence of medical pathologies potentially confounding the assessment of endometriosis-specific symptoms (e.g. cancer) were subsequently excluded from the analyses.

Interested participants provided their informed consent for study participation and completed a socio-demographic and medical history schedule followed by self-report questionnaires assessing positive and negative body image and HRQoL in the context of endometriosis. Participants took ∼30 min to complete the survey. They did not receive any compensation for their participation. The study was conducted in accordance with the Declaration of Helsinki and approved by the Ethical Committee for Psychological Research of the University of Padova.

### Measures

#### Socio-demographic information and medical history

Socio-demographic information collected included participant’s sex, gender, age, education, height, weight, marital, and employment status. Participants also reported endometriosis-specific medical history, including the method of diagnosis (i.e. surgical, clinical, diagnostic imaging), specific type of endometriosis (i.e. superficial, ovarian, deep), time (in months) since diagnosis, number, and type of prior and current endometriosis treatments, and the presence or absence of a range of common endometriosis symptoms experienced, including pelvic pain, dysmenorrhea, dyspareunia, dyschezia.

#### Positive body image

The *Functionality Appreciation Scale* (FAS; Alleva *et al.*, 2017; Cerea *et al.*, 2021) is a 7-item self-report questionnaire investigating functionality appreciation on a 5-point Likert scale (ranging from 1 = *strongly disagree* to 5 = *strongly agree*). Higher scores reflect higher levels of functionality appreciation. The Italian version of the questionnaire proved to be highly reliable, with good internal consistency (McDonald’s ω = 0.89) and adequate test–retest reliability (*r *= 0.83 in women and *r *= 0.73 in men; Cerea *et al.*, 2021). In the current study, the internal consistency of the FAS score was excellent (McDonald’s ω = 0.89).

The *Body Appreciation Scale-2* (BAS-2; Tylka and Wood-Barcalow, 2015b; Casale *et al.*, 2021) is a self-report questionnaire composed of 10 items rated on a 5-point Likert scale, ranging from 1 (*never*) to 5 (*always*). The BAS-2 measures acceptance of one’s body, respect and care for the body, and protection of one’s body from unrealistic beauty standards. Higher scores indicate higher levels of body appreciation. The Italian version of the BAS-2 showed good internal consistency values (McDonald’s ω = 0.93 in women and McDonald’s ω = 0.89 in men; Casale *et al.*, 2021). In the present study, the internal consistency value for the BAS-2 score was good (McDonald’s ω = 0.95).

#### Negative body image

The *Body Image Scale* (BIS; Hopwood *et al.*, 2001; Cheli *et al.*, 2016) investigates body image distress. Originally developed for oncology populations, it has been widely applied in other chronic disease contexts (Hehenkamp *et al.*, 2007; McDermott *et al.*, 2015), including endometriosis (e.g. Pehlivan *et al.*, 2022; Calvi *et al.*, 2024). The BIS items address bodily changes due to illness treatment (e.g. ‘Have you been feeling the treatment has left your body less whole?’) or symptoms of the condition itself. In accordance with a previous study (Pehlivan *et al.*, 2022), we adapted the BIS for an endometriosis sample, substituting the word ‘disease’ with ‘endometriosis’ in the instruction of the questionnaire. The BIS measures emotional, cognitive, and behavioral components of body image through 10 items on a 4-point Likert scale (from 0=‘*not at all*’ to 3=‘*very much*’). Higher scores correspond to greater body image distress (Hopwood *et al.*, 2001). The Italian version of the BIS showed good psychometric properties (Cronbach’s α = 0.92; Cheli *et al.*, 2016). In our sample, the internal consistency of the BIS score was excellent (McDonald’s ω = 0.93).

#### HRQoL in endometriosis

The *Endometriosis Health Profile-30* (EHP-30; Jones *et al.*, 2001; Gioia *et al.*, 2023) is a disease-specific scale measuring HRQoL in women with endometriosis. It consists of two parts: a core questionnaire and a modular questionnaire. The core questionnaire is applicable to all women with endometriosis and is composed of 30 items on a 5 points Likert scale (from 0=‘*never*’ to 4=‘*always*’), clustered into 5 subscales: pain (11 items), control and powerlessness (6 items), emotional well-being (6 items), social support (4 items), and self-image (3 items). The modular questionnaire consists of 23 items on a 5 points Likert scale (from 0=‘*never*’ to 4=‘*always*’) and it is related to 6 areas: work life (5 items), relationship with children (2 items), sexual intercourse (5 items), medical profession (4 items), treatment (3 items), and infertility (4 items). This modular questionnaire enables women to respond only to scales which are relevant to them (i.e. some parts of the questionnaire are not applicable to all women, such as for those who have no children). High scores reflect high levels of disability.

The Italian version of the EHP-30 (Gioia *et al.*, 2023) showed high internal consistency values, with Cronbach’s alpha coefficients ranging from 0.60 to 0.95 (core questionnaire) and 0.74–0.94 (modular questionnaire). In the current study, McDonald’s ω ranged from 0.84 (self-image) to 0.97 (pain).

### Data analysis

First, LPA was employed to identify distinct profiles of women with endometriosis based on body image dimensions. In particular, indicator variables covered positive body image (i.e. standardized scores of the BAS-2 and the FAS), negative body image related to chronic illnesses (i.e. standardized score of the BIS), and endometriosis-specific negative body image (i.e. standardized score of the EHP-30 self-image scale).

LPA models were established through a series of steps, starting with one-class model and gradually increasing the number of classes until no further improvement in model fit was observed (Lubke and Muthén, 2007). Model selection criteria included evaluating fit statistics, entropy values, profile size, and interpretability. As regards model fit statistics, the Bayesian Information Criterion (BIC), Akaike Information Criterion (AIC), and sample-size adjusted BIC (SABIC) were evaluated, with lower values indicating superior relative fit (Nylund *et al.*, 2007). The bootstrapped likelihood ratio test (BLRT) was also taken into account, with a significant value suggesting a better fit of the estimated model compared to a model with one less latent profile. Moreover, entropy was scrutinized, with values above 0.80 considered preferable (Muthén, 2001). In addition, the size of the smallest class was evaluated, and models with profiles representing less than 5% of the sample were rejected (Nylund-Gibson and Choi, 2018). The LPA was performed using the mclust (Scrucca *et al.*, 2016) and tidyLPA (Rosenberg *et al.*, 2018) R packages.

Subsequently, possible disparities in different HRQoL dimensions relevant to endometriosis, months since diagnosis, and total number of endometriosis symptoms were investigated by profile. To this end, Welch’s ANOVA – which adjusts the degrees of freedom to account for heterogeneity of variance across groups – was carried out. The EHP-30 emotional well-being, social support, physical pain, and control-powerlessness scales, along with months since diagnosis and number of endometriosis symptoms were considered as dependent variables. Games–Howell *post hoc* tests were chosen for pairwise comparisons due to the violation of homoscedasticity assumption. Chi-square (*χ*^2^) tests were then performed to explore the associations between profile membership and variables such as specific endometriosis diagnosis, endometriosis treatment, and type of endometriosis-related symptoms (i.e. non-painful symptoms due to menstrual cycle alterations, painful symptoms, and a-specific symptoms).

Importantly, months since diagnosis and endometriosis diagnosis and treatment were included in the analyses solely to ensure that the observed profile differences were not attributable to these variables. ANOVA and chi-square tests were conducted using the Jamovi statistical software (The Jamovi Project, 2022).

## Results

### Descriptive statistics of the sample

Table 1 reports the detailed socio-demographic and endometriosis-related characteristics of the sample. Table 2 instead shows the mean scores obtained by the participants on the administered questionnaires.

**Table 1.** Socio-demographic and endometriosis-related features of the sample (N = 270).

	Percentage (%)
Employment status	
Full-time	44.0
Part-time	17.8
Fixed term	8.9
Unemployed	7.4
Student	6.7
Housewife	5.2
Unable to work due to disability	1.1
Other	8.9
Marital status	
Married/domestic relationship	64.1
Engaged/Non-domestic relationship	13.3
Single	17.1
Separated/Divorced	4.1
Widow	0.7
Other	0.7
Method of endometriosis diagnosis	
Clinical	70.7
Surgical	22.6
Other	6.7
Endometriosis diagnosis	
Superficial endometriosis	5.2
Ovarian endometriosis	23
Deep endometriosis	58.1
Other type	7.0
Unknown type	6.7
Endometriosis treatment	
Hormonal medication	31.5
Surgery	7.0
Hormonal medication and surgery	54.4
Other treatments	7.0
Endometriosis symptoms	
Abdominal bloating	83.9
Pelvic pain	65.3
Reduced sexual desire	65.1
Dyspareunia	61.9
Headache	59.4
Dysmenorrhea	44.7
Dyschezia	40.1
Weight gain	37.6
Vulvodynia	32.6
Menstrual spotting	32.0
Menorrhagia	25.3
Irregular periods	22.0
Metrorrhagia	17.2

**Table 2.** Mean scores of the sample on the administered questionnaires.

	Mean	Standard Deviation
BAS-2	30.4	8.83
FAS	25.4	5.52
BIS	17	8.54
EHP-30—self-image	59.2	26.7
EHP-30—control/powerlessness	57.4	28.2
EHP-30—pain	47	26.1
EHP-30—emotional well-being	54.2	22.3
EHP-30—social support	60.5	25.7

*Note*. BAS-2 = Body Appreciation Scale-2; FAS=Functionality Appreciation Scale; BIS=Body Image Scale; EHP-30 = Endometriosis Health Profile-30.

### LPA based on body image-related dimensions

1- to 8-profile solutions were evaluated (Table 3), as the BLRT was no longer significant for the 9-profile model. First, the results showed that models with latent profiles fitted the data better compared to a solution without latent profiles. Examination of fit indices showed that the BIC favored the 5-profile model, while the AIC and SABIC supported the 8-profile model. However, solutions with 7 and 8 classes were all rejected due to the smallest class accounting for less than 5% of cases. Moreover, models with four to six profiles presented entropy values falling below the threshold of 0.80, so they were excluded from possible solutions. Therefore, the remaining 2- and 3-profile solutions were more closely investigated.

**Table 3.** Fit indices for the Latent Profile Analysis (LPA).

Model	AIC	BIC	SABIC	Entropy	Smallest profile (%)	BLRT *P*
1-profile	3077	3106	3080	1	1	
2-profile	2706	2752	2711	0.87	38.9	0.01
3-profile	2614	2679	2622	0.83	17	0.01
4-profile	2573	2655	2582	0.78	14.4	0.01
5-profile	2548	2649	2560	0.78	5.93	0.01
6-profile	2540	2659	2554	0.77	5.93	0.02
7-profile	2514	2651	2530	0.78	2.22	0.01
8-profile	2506	2661	2524	0.76	1.48	0.02
9-profile	2502	2674	2522	0.79	1.48	0.09

*Note.* AIC=Akaike Information Criterion; BIC=Bayesian Information Criterion; SABIC=sample-size adjusted BIC; Smallest profile (%)=percentage of individuals in the smallest profile; BLRT=bootstrapped likelihood ratio test.

Although the 2-profile model provided the best entropy value, 2 classes were deemed insufficient to capture the potential complexity of the configurations arising from the dimensions considered, resulting in the rejection of this solution. Instead, the 3-profile solution had the best-fit indices, and the entropy value was acceptable; additionally, each profile accounted for at least 5% of the sample and offered meaningful and interpretable distinctions. Based on these considerations, the 3-profile solution was considered the optimal model for explaining the data.

### Characteristics of the profiles and associations with endometriosis diagnosis, endometriosis treatment, and type of endometriosis-related symptoms

**Figure 1.**
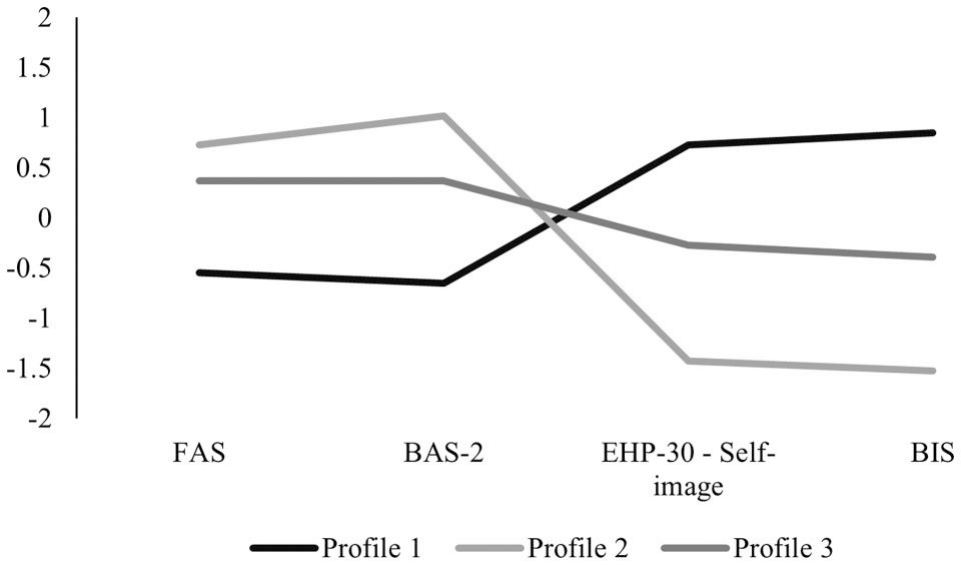
**Three-profile solution based on the standardized scores on the Body Appreciation Scale-2 (BAS-2), Functionality Appreciation Scale (FAS), self-image scale of the Endometriosis Health Profile-30 (EHP-30), and Body Image Scale (BIS).**  *Note*. Profile 1=‘Low body appreciation and strong body dissatisfaction’; Profile 2=‘Strong body appreciation and low body dissatisfaction’; Profile 3=‘Moderate body appreciation and body dissatisfaction’.

Figure 1 illustrates the three profiles based on the standardized scores on the BAS-2, FAS, BIS, and EHP-30 self-image scale. As can be noticed, each profile exhibited a distinct trend across these measures. In particular, the first profile included the largest proportion of participants (n = 129, 47.8%) and was characterized by low scores on the BAS-2 and the FAS, coupled with high scores on the BIS and the EHP-30 self-image scale; hence the label ‘Low body appreciation and strong body dissatisfaction’. The second profile comprised the smallest number of individuals (n = 46, 17%) and showed an opposing trend to the previous profile, that is high scores on the BAS-2 and FAS and low scores on the BIS and EHP-30 self-image scale; as a result, it was named ‘Strong body appreciation and low body dissatisfaction’. Finally, the third profile was featured by average scores on all the scales (n = 95, 35.2%) and thus called ‘Moderate body appreciation and body dissatisfaction’.

Tables 4 and 5 provide additional details regarding the endometriosis-related characteristics of women within each profile. Chi-square tests revealed no significant associations between profile membership and specific endometriosis diagnosis (*χ*^2^ _(8)_ = 10, *P *= 0.26), endometriosis treatment (*χ*^2^ _(6)_ = 10.9, *P *= 0.09), and the presence of non-painful symptoms due to menstrual cycle alterations (*χ*^2^ _(2)_ = 5.85, *P *= 0.054). Conversely, significant associations emerged for painful (*χ*^2^ _(2)_ = 22.2, *P *< 0.001) and a-specific symptoms (*χ*^2^ _(2)_ = 9.84, *P *= 0.007). In particular, although most women across all profiles reported experiencing painful and a-specific symptoms, the percentage difference between those who did and did not report these types of symptoms was significantly greater among participants in the ‘Low body appreciation and strong body dissatisfaction’ profile (Profile 1) (Table 5). In other words, participants in this profile were more likely to experience painful and/or a-specific symptoms compared to those in the other profiles.

**Table 4.** Endometriosis-related clinical variables of individuals in each profile.

Profile			
Variable	Low body appreciation and strong body dissatisfaction (n=129)	Strong body appreciation and low body dissatisfaction (n=46)	Moderate body appreciation and body dissatisfaction (n=95)
Endometriosis diagnosis (%)			
Superficial endometriosis	5.4	4.3	5.3
Ovarian endometriosis	20.2	32.6	22.1
Deep endometriosis	62.8	47.8	56.8
Unknown type	3.9	13	7.4
Other type	7.7	2.3	8.4
Treatment (%)			
Hormonal medication	31.8	19.6	36.8
Surgery	3.1	13	9.5
Hormonal medication and surgery	56.6	60.9	48.4
Other treatments	8.5	6.5	5.3

**Table 5.** Type of endometriosis-related symptoms reported by participants in each profile.

Profile			
Variable	Low body appreciation and strong body dissatisfaction (n=129)	Strong body appreciation and low body dissatisfaction (n=46)	Moderate body appreciation and body dissatisfaction (n=95)
Non-painful symptoms due to menstrual cycle alterations (%)			
Yes	55.5	34.8	48.4
No	44.5	65.2	51.6
Painful symptoms (%)			
Yes	93.8	65.2	83.2
No	6.3	34.8	16.8
A-specific symptoms (%)			
Yes	97.7	84.8	90.5
No	2.3	15.2	9.5

*Note.* Non-painful symptoms due to menstrual cycle alterations: menorrhagia, metrorrhagia, menstrual spotting, and irregular periods; painful symptoms: pelvic pain, dyspareunia, dyschezia, dysmenorrhea, and vulvodynia; a-specific symptoms: weight gain, reduced sexual desire, abdominal bloating, and headache.

### Differences between profiles in HRQoL dimensions relevant to endometriosis, months since diagnosis, and endometriosis-related symptoms

Table 6 presents the results of Welch’s ANOVA with profile membership as an independent variable and the EHP-30 emotional well-being, social support, physical pain, and control-powerlessness scales (i.e. HRQoL dimensions), along with months since diagnosis and total number of endometriosis-related symptoms as outcome variables. Significant differences were identified on all the EHP-30 scales, as well as in the total number of endometriosis-related symptoms. In particular, *post hoc* tests revealed that people in the ‘Low body appreciation and strong body dissatisfaction’ profile (Profile 1) exhibited significantly higher scores on all the EHP-30 subscales and the highest mean number of endometriosis-related symptoms compared to those in the other profiles.

**Table 6.** Results of ANOVA with profile membership as an independent variable.

		Profile 1	Profile 2	Profile 3	
	*F* (df1, df2)	*M*±SD	*M*±SD	*M*±SD	*Post hoc* comparisons
EHP-30—emotional well-being	57.1 (2, 112)*	65.1±16.5	28.9±21.1	51.1±19.3	1>3>2*
EHP-30—social support	73.3 (2, 104)*	74.6±15.4	32.5±24.2	54.8±24.6	1>3>2*
EHP-30—pain	15.8 (2, 113)*	55.6±23	33±29.3	41.9±24.4	1>2*, 1>3*
EHP-30—control/powerlessness	45.3 (2, 113)*	71.1±22.2	31.4±28.2	51.3±24.4	1>3>2*
Time (months) since diagnosis	0.43 (2, 121)	342.9±73.1	347.5±74.7	353.3±91	–
Number of endometriosis-related symptoms	29.3 (2, 123)*	6.75±2.62	3.53±2.36	5.52±2.49	1>3>2*

*Note.* Profile 1=‘Low body appreciation and strong body dissatisfaction’; Profile 2=‘Strong body appreciation and low body dissatisfaction’; Profile 3=‘Moderate body appreciation and body dissatisfaction’; EHP-30 = Endometriosis Health Profile-30, df=degrees of freedom, *M*=mean.

* *P *< 0.001.

Conversely, individuals in the ‘Strong body appreciation and low body dissatisfaction’ profile (Profile 2) showed significantly lower EHP-30 scores and the lowest mean number of endometriosis-related symptoms. However, on the EHP-30 pain scale, women in the ‘Strong body appreciation and low body dissatisfaction’ profile (Profile 2) did not significantly differ from those in the ‘Moderate body appreciation and body dissatisfaction’ profile (Profile 3). Finally, no significant differences between profiles were observed concerning months since diagnosis (*P *= 0.66).

## Discussion

The present study mainly aimed to pinpoint meaningful profiles of body image in a group of Italian women with endometriosis. Moreover, these profiles were compared in terms of HRQoL dimensions specific to endometriosis and number and types of endometriosis-related symptoms, while controlling for months since diagnosis, specific endometriosis diagnosis, and treatment type.

First, LPA identified three distinct profiles: ‘Low body appreciation and strong body dissatisfaction’, ‘Strong body appreciation and low body dissatisfaction’, and ‘Moderate body appreciation and body dissatisfaction’. Women in the ‘Low body appreciation and strong body dissatisfaction’ profile reported significant dissatisfaction and distress with their body, and minimal appreciation for their body and its functionality. At the opposite end, the ‘Strong body appreciation and low body dissatisfaction’ profile was indicative of a more favorable body image configuration, as women in this group seem to display a healthier and more positive relationship with their body. Finally, women in the ‘Moderate body appreciation and body dissatisfaction’ profile simultaneously experienced moderate levels of body dissatisfaction and body and functionality appreciation.

Regarding the trend observed in the ‘Moderate body appreciation and body dissatisfaction’ profile, it resonates with previous LPA studies involving community adult (Jackson *et al.*, 2022; Raspovic *et al.*, 2023) and adolescent (Iannattone *et al.*, 2024) samples, which have similarly identified subgroups of individuals characterized by the simultaneous presence of both negative and positive body image. This finding also aligns with a well-established body of literature recognizing that negative and positive body image are conceptually distinct constructs that can co-occur (Halliwell, 2015; Tylka and Wood-Barcalow, 2015a; Bailey *et al.*, 2016; Alleva and Tylka, 2021), also in populations with chronic illness (Markey *et al.*, 2020; Moro *et al.*, 2024). Although the coexistence of negative and positive body image is not theoretically novel, including in the context of chronic illnesses, the current study offers a valuable contribution by empirically documenting this coexistence for the first time within a sample of women with endometriosis.

Crucially, most participants were classified into the ‘Low body appreciation and strong body dissatisfaction’ profile, while the ‘Strong body appreciation and low body dissatisfaction’ profile included the smallest percentage of women. These findings align with the literature showing the centrality of body image-related issues for women with endometriosis (e.g. Van Niekerk *et al.*, 2022b; Mills *et al.*, 2025). However, this picture is concerning, as, in line with our hypotheses, the ‘Low body appreciation and strong body dissatisfaction’ profile was more compromised in terms of HRQoL than other profiles, being specifically characterized by marked feelings of lack of control and powerlessness, reduced social support, and elevated physical pain and emotional distress. Furthermore, women in this profile reported the highest mean number of endometriosis symptoms and were also more likely to experience painful and a-specific symptoms. Nevertheless, although the majority of participants in our study were classified into this profile, the absence of a control group of women without endometriosis prevents us from claiming that the identified body image profile is exclusive to women with endometriosis.

Conversely, the ‘Strong body appreciation and low body dissatisfaction’ profile seems to reflect a more favorable physical and psychological state, associated with better HRQoL and fewer and less impactful endometriosis-related symptoms. Although our analysis cannot definitely establish the directionality of the relation between body image configurations, HRQoL, and endometriosis symptoms, the present findings suggest that body image may play an important role in shaping HRQoL and physical well-being. Particularly, in keeping with previous variable-centered evidence (Melis *et al.*, 2015; Van Niekerk *et al.*, 2022a), it is plausible that negative body image in women with endometriosis may represent a risk factor for impaired HRQoL and heightened perception of endometriosis symptoms. Indeed, women who perceive their body negatively might be more vulnerable to physical discomfort, social difficulties, psychological distress, and heightened symptom perception, which could exacerbate the overall burden associated with endometriosis and its treatment.

On the contrary, positive body image may act as a protective factor, possibly helping to buffer against the adverse impacts of endometriosis on various dimensions of HRQoL and physical well-being (Sayer-Jones and Sherman, 2021, 2023; Volker and Mills, 2022). In this regard, women with high levels of positive body image may experience greater emotional resilience, perceived stronger social support, and report better physical and psychological functioning, all of which can contribute to a more adaptive adjustment to the challenges posed by the illness and its management.

That said, it is also possible that poor HRQoL, alongside elevated and distressing endometriosis symptoms, affects body image. Indeed, women’s lived experiences with reduced HRQoL, shaped by the burden of persistent and often painful symptoms, may negatively influence how they perceive and feel about their body (Markey *et al.*, 2020). In this sense, negative body image may also reflect the physical and psychological burden of endometriosis itself. For instance, body-related limitations introduced by endometriosis and its treatment (La Rosa *et al.*, 2020; Mills *et al.*, 2025) may hinder the development of adaptive and holistic ways to appraise the body (Markey *et al.*, 2020). Moreover, as endometriosis affects multiple dimensions of HRQoL, these compounded stressors may erode women’s ability to appreciate their body, further distancing them from positive body image. Thus, future research should explore the interplay between body image, HRQoL, and physical well-being in women with endometriosis, as a more comprehensive understanding of this dynamic may help clarify how these factors influence each another.

Finally, no significant differences were found between the ‘Strong body appreciation and low body dissatisfaction’ and ‘Moderate body appreciation and body dissatisfaction’ profiles regarding reduced HRQoL due to physical pain. Specifically, and surprisingly, women in the ‘Moderate body appreciation and body dissatisfaction’ group, despite experiencing more body dissatisfaction and less body and functionality appreciation, did not report significantly greater impairment in HRQoL related to chronic pain. A possible explanation is that even moderate levels of positive body image may offer some degree of psychological resilience and protection against the negative effects of endometriosis-related pain on HRQoL, potentially approaching the benefits associated with higher levels of positive body image.

However, the reverse relation is also plausible: impaired HRQoL due to physical pain may influence body image (Markey *et al.*, 2020). In particular, it could be that low or moderate levels of physical pain-related impairment in HRQoL produce similar effects on body image. Instead, the most detrimental effects on body image may emerge when the impairment in HRQoL due to physical pain becomes more severe. Intense pain, by disrupting daily functioning (Hunsche *et al.*, 2023), can exacerbate negative body image and a sense of disconnection from the body. Simultaneously, high levels of physical pain may diminish a woman’s ability to appreciate her body, as it becomes seen primarily as a source of pain and limitation (Markey *et al.*, 2020). This suggests that the impact of physical pain on HRQoL could play a meaningful role in shaping body image in women with endometriosis, with severe impairments potentially promoting negative body image and reducing positive body image. This perspective underscores the importance of considering the severity of physical pain and its effects on both HRQoL and body image, particularly in populations with chronic conditions such as endometriosis.

To conclude, it is important to note that the body image profiles identified in this study were not significantly related to treatment type, months since diagnosis, or the specific subtype of endometriosis diagnosis. This finding suggests that body image experiences in women with endometriosis are not directly tied to clinical indicators of illness severity. In other words, whether a woman has been recently diagnosed or has lived with endometriosis for many years, and regardless of the specific type of endometriosis or treatment she is receiving, the patterns of positive and negative body image appear to operate independently of these medical factors. This result is particularly important, as it underscores that body image in this population may be shaped by broader psychological, social, or contextual factors rather than merely by the progression of the disease. Further research is needed to investigate which factors may contribute to the development of negative or positive body image among women with endometriosis.

Although this study provided novel findings, several limitations must be acknowledged, suggesting avenues for future research. First, the study’s cross-sectional design prevents any causal inferences from being drawn. Thus, the present results offer only preliminary insights into how negative and positive body image may influence each other and HRQoL in women with endometriosis. To better understand the development and progression of body image configurations and their outcomes, future research should employ longitudinal designs. Indeed, by monitoring these profiles over time, researchers could identify key periods when women with endometriosis are more susceptible to negative body image or, conversely, more inclined to appreciate their body and its functionality.

Second, the current study lacks a control group of individuals without endometriosis. The inclusion of a control group would enable comparisons between women with endometriosis and those without the condition, offering a clearer understanding of how the relations between body image profiles and HRQoL are uniquely affected by the illness. Without such a comparison, it remains unclear whether the identified body image profiles are specific to women with endometriosis or whether they reflect broader patterns in body image across the general population. However, this comparison was not feasible within the current study design, as the instruments used to assess negative body image—such as the EPH-30, which is disease-specific, and the BIS, which targets individuals with chronic conditions—are not applicable to individuals without endometriosis. As such, our focus remained on capturing the nuances of body image within the context of this specific clinical population.

Third, it is important to acknowledge that a small number of participants (n = 4) reported having sought psychological consultation for a possible eating disorder. Given the study’s focus on body image, this could be viewed as a potential source of bias. However, preliminary sensitivity analysis excluding these individuals revealed no substantive differences in the results. Therefore, they were retained in the final sample to reflect the natural diversity of women with endometriosis and to capture a broader spectrum of body image experiences within this population.

Fourth, we did not collect data on participants’ sexual orientation. This omission may limit the generalizability of our findings, as it prevented us from capturing potentially important variability in the experiences of endometriosis and body image across diverse groups. Future research should incorporate sexual orientation to ensure more comprehensive and representative analyses. In addition, in future studies, it is crucial to involve women from diverse social and cultural backgrounds to evaluate the generalizability of the identified profiles across different nations and cultures.

The generalizability of the findings may also be limited by the recruitment method, as self-referral through endometriosis organizations may have resulted in a sample biased toward individuals who are more severely affected by or more concerned about their condition. Future research should aim to include individuals recruited through a broader range of settings to enhance the representativeness of the sample.

Another notable limitation of the current study is its exclusive use of self-report questionnaires, which are prone to biases (e.g. social desirability, memory recall, and varying interpretations of questions). Therefore, future studies should consider combining data from multiple sources, including qualitative methods such as semi-structured interviews and focus groups. This mixed-methods approach would enrich the understanding of body image issues in women with endometriosis by offering more nuanced and detailed perspectives on their lived experiences.

Finally, future studies should measure other variables theoretically related to body image and HRQoL, such as resilience, embodiment, intuitive eating, interoceptive awareness, and physical activity. At the same time, they should measure other psychological (e.g. personality traits), behavioral (e.g. sexual activity), and biological (e.g. hormonal levels) variables relevant in the context of endometriosis, to elucidate underlying mechanisms contributing to distress in women with this condition.

## Conclusion and practical implications

Despite the aforementioned shortcomings, the study contributes to the growing body of research on body image in the context of endometriosis. Indeed, the identification of three distinct body image profiles, each with unique relations with HRQoL dimensions and both the number and types of endometriosis-related symptoms, represents a starting point in understanding the interplay between these factors in women with this health condition. To be specific, the co-occurrence of negative and positive body image underscores the complexity of body image dynamics in women with endometriosis, suggesting that focusing solely on one aspect (i.e. negative or positive body image) may not be sufficient to fully capture the multifaceted experiences of body image and their potential impact on HRQoL in this population. The practical implications of these results may be meaningful, as uncovering how different body image profiles relate to HRQoL and symptom burden might inform both case conceptualization and the development of psychological interventions tailored to women with different medical profiles of endometriosis.

Specifically, for case conceptualization, it may be important for clinicians to assess not only the presence and severity of physical symptoms but also patients’ relationship with their body, as this aspect could contribute to their adaptation to the illness. Moreover, from a treatment perspective, interventions aimed at enhancing body and functionality appreciation might represent a promising avenue for reducing endometriosis-related impairments in HRQoL and mitigating the adverse effects of the illness’ symptoms. Such approaches may be especially beneficial for women reporting painful symptoms (e.g. pelvic pain, dyschezia, etc) and/or a-specific symptoms (e.g. headache, weight gain, etc), as they may experience more pronounced impairments in body image. In conclusion, these findings suggest the potential value of integrating body image considerations into both clinical assessment and treatment planning, ensuring that psychological and medical care addresses the multifaceted challenges faced by women with endometriosis.

## Data Availability

The anonymized dataset and R code used for LPA are available at the following OSF page: https://osf.io/c4ve7/?view_only=f6ed7b76e5614cf580846f07f5b4c117
